# Pyruvate dehydrogenase complex plays a central role in brown adipocyte energy expenditure and fuel utilization during short-term beta-adrenergic activation

**DOI:** 10.1038/s41598-018-27875-3

**Published:** 2018-06-22

**Authors:** Ntsiki M. Held, Eline N. Kuipers, Michel van Weeghel, Jan Bert van Klinken, Simone W. Denis, Marc Lombès, Ronald J. Wanders, Frédéric M. Vaz, Patrick C. N. Rensen, Arthur J. Verhoeven, Mariëtte R. Boon, Riekelt H. Houtkooper

**Affiliations:** 1 Laboratory Genetic Metabolic Diseases, Academic Medical Center, 1105 AZ Amsterdam, The Netherlands; 2 Department of Medicine, Division of Endocrinology, Leiden University Medical Center, 2333 ZA Leiden, The Netherlands; 3 Einthoven Laboratory of Experimental Vascular Medicine, Leiden University Medical Center, 2333 ZA Leiden, The Netherlands; 4 Department of Human Genetics, Leiden University Medical Center, 2333 ZA Leiden, The Netherlands; 5 Institut National de la Santé et de la Recherche Médicale, Unité 1185, UMR_S 1185, Fac Med Paris Sud, Université Paris-Saclay, Le Kremlin-Bicêtre, France

## Abstract

Activation of brown adipose tissue (BAT) contributes to total body energy expenditure through energy dissipation as heat. Activated BAT increases the clearance of lipids and glucose from the circulation, but how BAT accommodates large influx of multiple substrates is not well defined. The purpose of this work was to assess the metabolic fluxes in brown adipocytes during β3-adrenergic receptor (β3-AR) activation.T37i murine preadipocytes were differentiated into brown adipocytes and we used Seahorse respirometry employing a set of specific substrate inhibitors in the presence or absence of β3-AR agonist CL316,243. The main substrate used by these brown adipocytes were fatty acids, which were oxidized equally during activation as well as during resting condition. [U-^13^C]-glucose tracer-based metabolomics revealed that the flux through the TCA cycle was enhanced and regulated by pyruvate dehydrogenase (PDH) activity. Based on ^13^C-tracer incorporation in lipids, it appeared that most glucose was oxidized via TCA cycle activity, while some was utilized for glycerol-3-phosphate synthesis to replenish the triglyceride pool. Collectively, we show that while fatty acids are the main substrates for oxidation, glucose is also oxidized to meet the increased energy demand during short term β3-AR activation. PDH plays an important role in directing glucose carbons towards oxidation.

## Introduction

One of the major health threats of today’s society is obesity. Obesity develops as a consequence of a long-term positive energy balance and is associated with the onset and progression of dyslipidaemia, type 2 diabetes, cardiovascular disease, and certain types of cancer^1^. Brown adipose tissue (BAT) in adult humans is involved in non-shivering thermogenesis and thereby contributes to whole body energy expenditure^2–5^. The functional relevance of BAT activity in adult humans has been underscored in recent years as repeated BAT activation through cold exposure reduces body fat^6^, and improves insulin sensitivity in lean^7,8^, obese^9^ and type 2 diabetic individuals^10^. BAT activation is therefore regarded as a novel therapy to treat obesity and related metabolic disorders^11^.

The therapeutic potential of BAT activation originates from its potent metabolic oxidative capacity that is due to a high number of mitochondria that express uncoupling protein 1 (UCP1). UCP1 activity allows BAT mitochondria to uncouple respiration from ATP production and generate heat instead^12^. Physiologically, this thermogenic function is induced by cold exposure which results in enhanced sympathetic outflow towards brown adipocytes and binding of noradrenaline to the β3-adrenergic receptor (β3-AR) on brown adipocytes. BAT activation induces the release of internally stored substrate pools as well as the uptake of vast amounts of circulating glucose and lipids^13–17^. Despite the large influx of glucose in activated brown adipocytes, fatty acids are suggested to be the preferred substrates during thermogenesis^12^, in line with high fatty acid oxidation upon BAT activation in mice^16^ and in humans^18^. These fatty acids are released by lipolysis but also originate from the uptake of circulating lipids. Interestingly, inhibition of lipolysis reduces the uptake of glucose and lipids significantly and results in a dampened thermogenic response^15,17^. It has therefore been suggested that substrate uptake is high in activated brown adipocytes to replenish the intracellular lipid pool^17,19^. This metabolic effect in BAT has primarily been studied through PET-CT tracer studies and gene expression arrays, which counterintuitively showed concomitant increased expression of glycolysis, β-oxidation, glycogen and fatty acid synthesis gene^13,17,20–22^. This implies that substrate utilization in BAT is regulated in a different way as compared to that of liver and muscle, which typically follow the principles of the Randle or glucose-fatty acid cycle. These principles are based on the idea that substrates compete for their oxidation due to inhibitory effects of intermediate metabolites^23^. For example, during fatty acid oxidation the levels of acetyl-CoA increase. The rise of this intermediary metabolite has an inhibitory effect on enzymes involved in glucose oxidation. The BAT-specific regulation allowing simultaneous uptake, storage and oxidation of glucose and fatty acids is poorly understood.

In this study, we used the T37i murine brown adipocyte cell line to determine metabolic fluxes of the most common substrates glucose, fatty acids and glutamine during short-term β3-AR activation. We used a set of specific inhibitors to selectively inhibit either the uptake or oxidation of substrates and determined the contribution of these substrates to cellular oxygen consumption using Seahorse respirometry. Furthermore, we applied ^13^C-stable isotope tracer-based metabolomics to examine the detailed metabolic wiring in these brown adipocytes. We found that pyruvate dehydrogenase plays a central role in directing glucose to oxidative metabolism during acute activation in brown adipocytes, while glucose is also utilized to replenish the intracellular triglyceride pool after long-term stimulation.

## Results

### T37i brown preadipocytes acquire a BAT bioenergetic profile during differentiation

**Figure 1:**
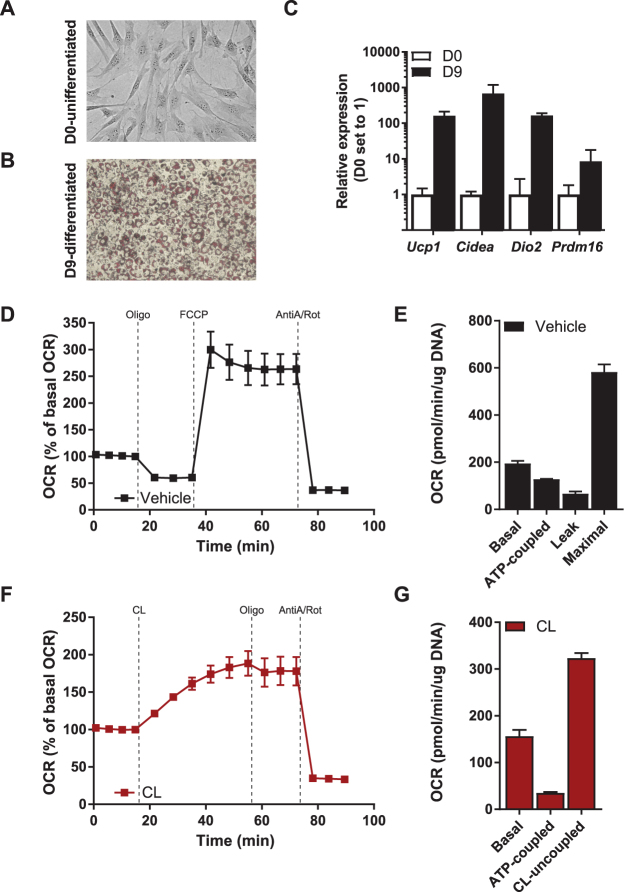
T37i bioenergetic profile during differentiation and β3-AR stimulation. **(A,B)** Oil-Red-O stained phase contrast images of undifferentiated **(A)** and differentiated **(B)** T37i cells. **(C)** Induction of mRNA expression of brown adipocyte genes after differentiation at day 9 relative to expression at day 0. **(D)** Oxygen consumption rate (OCR) of differentiated cells and **(E)** quantification of basal respiration, ATP-coupled and leak respiration after successive addition of 1.5 µM oligomycin, maximum respiration induced by 2 µM FCCP, corrected for non-mitochondrial respiration calculated after addition of 1.25 µM rotenone and 2.5 µM antimycin A. **(F)** OCR after CL316,243 and oligomycin addition and **(G)** quantification of basal, ATP coupled and CL-uncoupled respiration. Data is presented as average of three experiments ± SEM.

To characterize substrate utilization in brown adipocytes upon acute β3-adrenergic activation, we utilized the T37i brown preadipocyte cell line^24,25^. Prior to differentiation these cells have a fibroblast-like phenotype (Fig. 1A). T37i cells differentiated into brown adipocytes after at least nine days of exposure to insulin and triiodothyronine (T3). Successful differentiation was characterized by multilocular lipid droplets and induction of the thermogenic gene program (Fig. 1B,C), confirming the brown adipocyte phenotype.

Inducibility of the β3-adrenergic response serves as ultimate evidence of differentiation into mature brown adipocyte. To determine this, we used Seahorse respirometry to measure oxygen consumption rate (OCR) in the presence of vehicle or the β3-AR agonist CL316,243. First, we determined the mitochondrial bioenergetic profile by recording respiration under basal, ATP coupled, oligomycin-inhibited (leak) and maximal (uncoupled) conditions (Fig. 1D). Without β3-AR stimulation, the ATP-coupled and leak respiration represented 66% and 34% of the basal respiration rate, respectively. The chemical uncoupler FCCP induced maximal respiration to 334% of basal respiration (Fig. 1E). CL316,243 also induced uncoupled respiration but the response was less pronounced and acute compared to FCCP (Fig. 1F). CL316,243 reduced the ATP coupled respiration to 20% of basal OCR (Fig. 1G). These data confirm that T37i is a bona fide BAT model that displays a high mitochondrial activity and a robust β3-AR response^26^.

### β3-AR activation improves metabolic flexibility in brown adipocytes

Substrate availability and utilization dictate the mitochondrial respiratory capacity. Our first approach to determine substrate utilization under basal conditions and upon β3-AR activation was to measure respiration in the presence of glutamine oxidation, fatty acid oxidation and glucose oxidation inhibitors. The effect of a single substrate inhibitor on respiration determines how much this respiration is dependent on a given substrate—a parameter known as substrate dependency. Glutamine dependency was determined by the response to BPTES, an inhibitor of glutaminase^27^. Fatty acid oxidation dependency was estimated with POCA, a CPT1 inhibitor^28^. Finally, glucose oxidation dependency was determined by the sensitivity to UK5099, which is an inhibitor of the mitochondrial pyruvate carrier^29^. The substrate dependency was calculated as a percentage of the total reduction in OCR when all three inhibitors were administered.

**Figure 2:**
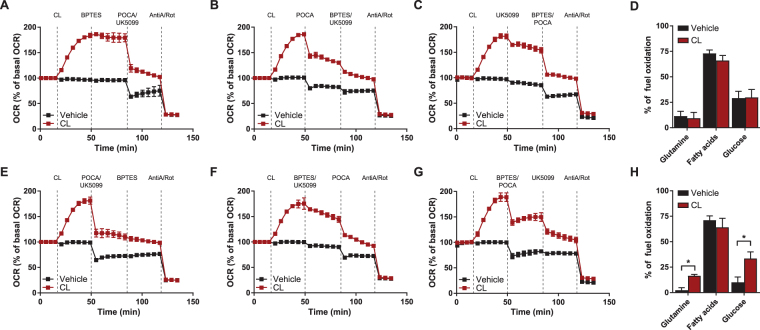
β3-AR activation improves metabolic flexibility in brown adipocytes. Representative OCR trace showing substrate dependence for maintaining respiration based on individual inhibitor addition strategies. Substrate oxidation dependence was determined for **(A)** glutamine with BPTES, **(B)** fatty acids with POCA and **(C)** glucose with UK5099. **(D)** Quantification of substrate dependence highlights that both resting and activated T37i adipocytes predominantly rely on fatty acid utilization to sustain OCR. Representative OCR trace showing **(E)** glutamine, **(F)** fatty acid and **(G)** glucose reserve capacity for maintenance of respiration after simultaneous addition of two inhibitors. **(H)** Quantification of substrate reserve capacity in resting and activated T37i cells. Glutamine and glucose oxidation reserved capacity is induced in CL316,243 stimulated cells. Line graphs show mean ± SD of 6 wells of one representative experiment. Data in bar graphs are presented as mean ± SEM of four experiments; *P < 0.05 (unpaired Student’s t-test).

When comparing these three substrates it was evident that addition of BPTES hardly affected the OCR and hence that glutamine oxidation contributes the least to mitochondrial respiration (Fig. 2A). The T37i brown adipocytes were most sensitive to inhibition of fatty acid oxidation, indicating that they primarily depend on fatty acid oxidation to maintain mitochondrial respiration (Fig. 2B). This strong reliance on fatty acid oxidation was not further induced by β3-AR activation. It is worth noting that the substrate dependency was not influenced by β3-AR stimulation, i.e. resting and activated cells rely predominantly on fatty acid oxidation, although the respiration rates were obviously higher after CL316,243 treatment (Fig. 2D).

Next, we treated the differentiated T37i cells with combinations of two of the above-mentioned inhibitors. The residual respiration after the addition of two inhibitors marks the capacity to operate on a single substrate—a parameter known as reserve capacity (Fig. 2E**–**G). As these brown adipocytes depend most on fatty acid oxidation, this pathway also had the largest reserve capacity (Fig. 2H). This highlights that fatty acid oxidation could compensate for the loss of glutamine and glucose oxidation in both rest and activated state (Fig. 2H). The reserve capacity of glutamine oxidation was and that of glucose oxidation was marginal during rest (Fig. 2H,G). However, upon β3-AR stimulation the reserve capacity of these substrates, especially of glucose oxidation, was markedly induced (Fig. 2H). Taken together, this suggests that brown adipocytes strongly rely on fatty acid oxidation but are metabolically more flexible in their substrate utilization upon β3-AR activation.

### Increased glycolytic flux contributes to uncoupled respiration in activated brown adipocytes

**Figure 3:**
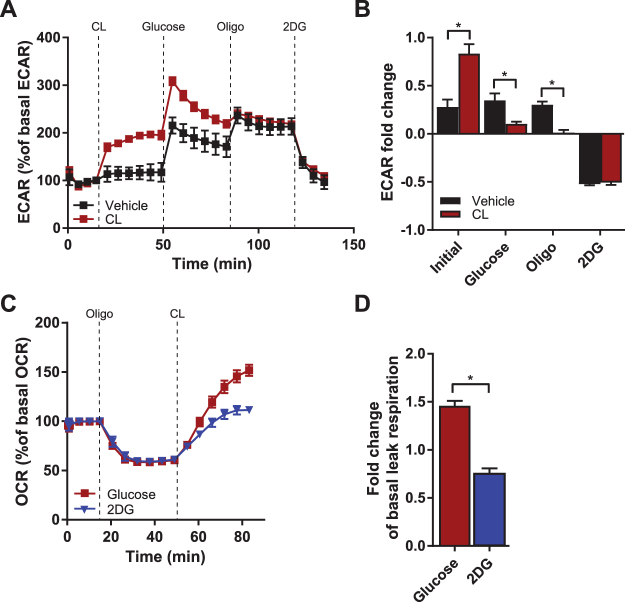
Increased glycolytic flux contributes to uncoupled respiration in activated brown adipocytes. **(A)** Extracellular acidification rate (ECAR) of vehicle and CL316,243-stimulated cells in response to 10 mM glucose, 1.5 µM oligomycin and 100 mM 2-deoxy-D-glucose (2DG). **(B)** ECAR fold change compared to basal ECAR shows increased initial glycolytic flux but unchanged glycolytic capacity. **(C)** Oxygen consumption rate (OCR) of cells under basal conditions, after addition of 1.5 µM oligomycin and 10 µM CL316,243 combined with 10 mM D-glucose (in red) or with 100 mM 2DG (in blue). **(D)** 2DG significantly reduces uncoupled respiration in activated brown adipocytes. Line graphs show a representative experiment with mean ± SD of 12 wells. Data in bar graphs are presented as mean ± SEM of three experiments; *P < 0.05 (unpaired Student’s t-test).

Despite the large influx of glucose during activation, glucose has been suggested to play a minor role in BAT oxidative capacity^12^. For the T37i cells, glucose was indeed the second preferred substrate, although activated cells could increase the oxidation of glucose markedly if necessary. To establish whether the reduction of ATP-coupled respiration after β3-AR activation (Fig. 1E,G) increases the glycolytic rate, we measured the extracellular acidification rate (ECAR) as an indicator of glycolysis. Indeed, addition of CL316,243 induced glycolysis maximally, as it could not be further induced by the addition of glucose or oligomycin (Fig. 3A,B). We next evaluated whether glycolysis contributes to uncoupled respiration by β3-AR stimulation. Uncoupled oxygen consumption without adrenergic activation was measured after addition of oligomycin, followed by addition of CL316,243 in combination with glucose or the glycolysis inhibitor 2-deoxy-glucose (2DG) (Fig. 3C). The capacity to induce uncoupled respiration was blunted when glycolysis was inhibited (Fig. 3D), supporting the notion that glucose utilization contributes to β3-AR induced uncoupled respiration in brown adipocytes.

### The glucose carbon flux into the TCA cycle is stimulated in β3-AR activated brown adipocytes

**Figure 4:**
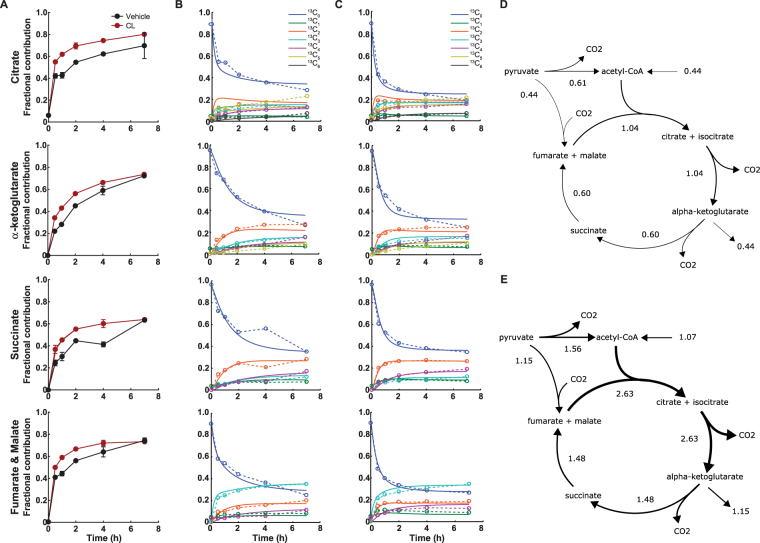
Increased glucose oxidation through TCA cycle activity after β3-AR activation. **(A)** Time course incubation with [U-^13^C]-glucose results in increased labelling of tricarboxylic acid (TCA) cycle intermediates at early time points after CL316,243 activation, suggesting increased TCA flux. **(B)** The measured MID data in dotted lines and predicted MID data in solid lines of vehicle-treated cells and **(C)** CL316,243-treated cells. **(D)** Results of non-stationary metabolic flux analysis of cells after vehicle and **(E)** β3-AR stimulation. The flux values have unit [/h] and are normalized to the alpha-ketoglutarate/glutamate concentration. Data represent mean ± SD of two separate experiments.

Fatty acids are an important carbon source of T37i cells, but upon β3-AR stimulation glucose uptake and glycolysis are greatly induced. Inhibition of glycolysis affects uncoupled respiration indicating that glucose is oxidized to contribute to uncoupled respiration (Fig. 3C,D). Gene expression studies show that short- and long-term cold-induced BAT activation increases expression of genes involved in glycolysis, the pentose phosphate pathway, fatty acid oxidation but also of fatty acid synthesis^20–22^. To assess through which pathways glucose is preferentially utilized during short-term β3-adrenerigic activation, we incubated cells with [U-^13^C]-glucose in the presence of CL316,243 or vehicle. We reconstructed metabolic flux distribution by measuring ^13^C-labeled metabolites using untargeted metabolomics after 30 min up to 7 h. First, we focused on labelling in glycolysis and pentose phosphate pathway (PPP) intermediates after acute activation. The PPP gives rise to NADPH that is required for lipid synthesis and glutathione antioxidant defence system, two processes suggested to be upregulated upon BAT activation^20^. Indeed,^13^C-labeling in PPP intermediates appeared to be increased after 30 min of β3-AR activation (Table S1). Possibly, the increased PPP activity in activated brown adipocytes caused the observed decrease in labelling in glycolytic intermediates (Table S1). However, after ≥2 h, labeling of glycolytic and PPP intermediates were equal in vehicle and CL316,243 treated cells (Table S1). Interestingly, despite the initial reduced label incorporation in pyruvate, TCA cycle intermediates were rapidly labelled following [U-^13^C]-glucose incubation. The rate of label incorporation in TCA cycle intermediates was greatly enhanced from 30 min up to 7 h after β3-AR stimulation (Fig. 4A). To estimate the flux through the TCA cycle we performed non-stationary flux analysis on the ^13^C label incorporation mass isotopomer distribution (MID) time patterns (Fig. 4B,C). Metabolic flux analysis showed that the overall TCA activity was increased upon β3-AR activation. Pyruvate entered the TCA cycle via pyruvate dehydrogenase (PDH) and pyruvate carboxylase (PC) activity which were both increased upon β3-AR activation (Fig. 4D,E). It appears that the overall flux of glucose metabolism is increased after β3-AR activation.

### Short-term β3 adrenergic activation stimulates pyruvate dehydrogenase activity without stimulating *de novo* lipogenesis

**Figure 5:**
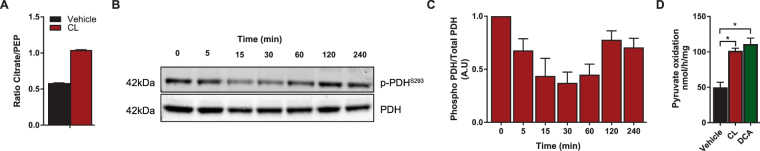
β3-AR stimulation increases glucose flux through PDH. **(A)** Increased ratio of ^13^C-labeled citrate over ^13^C-labeled phosphoenolpyruvate (PEP) after 30 min CL316,243 activation suggests increased flux through pyruvate dehydrogenase (PDH). **(B)** Western blot showing reduced PDH phosphorylation at serine-293, which is indicative for PDH activation. **(C)** The ratio of the quantified phosphorylated PDH E1-α serine-293 over the total PDH E1-α. **(D)** Upon 1 h CL316,243 stimulation pyruvate oxidation (using [1-^14^C]-pyruvate as substrate) is increased to a level that is similar as 1 h stimulation with the established PDH activator dichloroacetate (DCA). Data in **(A)** is mean ± SD of duplicate experiments, and **(B,C)** represent mean ± SEM of triplicates; *P < 0.05 (one-way ANOVA, Bonferroni corrected).

It has been suggested that glucose enters the TCA cycle merely to be exported to the cytoplasm as citrate to act as a building block for *de novo* lipogenesis^19^. Glucose oxidation is tightly regulated at the level of PDH, and increased PDH activity is associated with *de novo* lipogenesis^30,31^. Metabolic flux analysis estimated a 2.5-fold increase in PDH flux (Fig. 4D**–**E). To estimate this flux through PDH from our flux distribution measurements, we measured the ratio of ^13^C-labeled citrate to ^13^C-labeled phosphoenolpyruvate (PEP). This ratio was markedly increased in the CL316,243-activated cells which is indeed indicative of increased PDH flux. This allows carbons to enter the TCA cycle more rapidly in the activated state (Fig. 4A). The activity of PDH is regulated by phosphorylation and is influenced by many factors including mitochondrial redox status, intracellular ATP, pyruvate and acetyl-CoA levels^32^. The phosphorylated PDH is inactive, preventing pyruvate conversion to acetyl-CoA. Stimulation with CL316,243 resulted in a short-term dephosphorylation of PDH which peaked after 15–60 min of stimulation (Fig. 5B). Indeed, in this time window pyruvate oxidation rate was doubled (Fig. 5C), accounting for the strong glucose driven respiration after CL316,243 (Fig. 2C,G**–**H). Interestingly, 1 h stimulation with CL316,243 increased PDH activity to a similar extent as 1 h stimulation with the established PDH activator dichloroacetate (DCA), which activates PDH through the inhibition of PDH kinase^33^.

**Figure 6:**
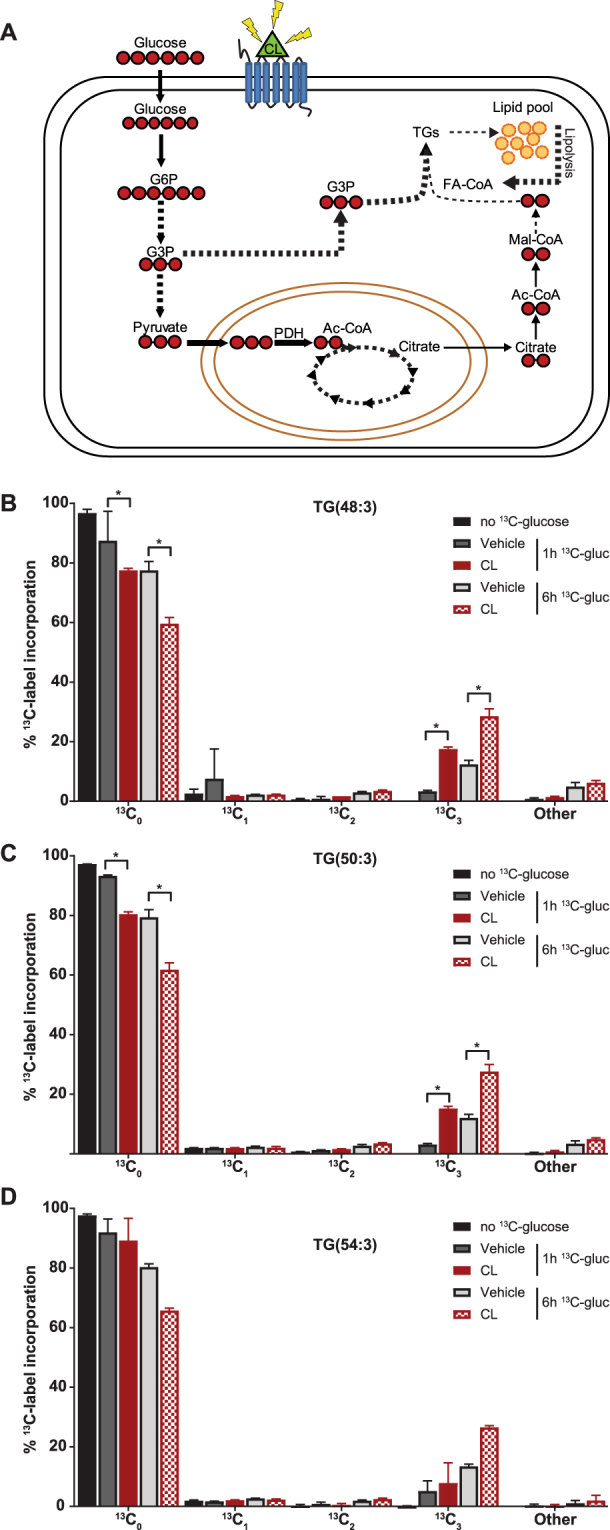
Short-term β3-AR stimulation increases fatty acid re-esterification. **(A)** Schematic illustration of label incorporation scenarios in lipids. Ac-CoA, acetyl-CoA; G6P, glucose-6-phosphate; G3P, glycerol-3-phosphate;FA-CoA, fatty acyl-CoA; mal-CoA, malonyl-CoA; PDH, pyruvate dehydrogenase. Solid lines and dashed lines indicate direct and indirect fluxes. Isotope profile of the three most abundant triglycerides **(B)** TG(48:3), **(C)** TG(50:3) and **(D)** TG(54:3) after 0, 1 and 6 hours of CL316,243 or vehicle stimulation. Incorporation of even carbons (^13^C_[n**×**2]_) is relatively low, while the ^13^C_3_ is increased over time suggesting glycerol incorporation in TG. CL316,243 induces glycerol incorporation significantly after 1 and especially after 6 hours. Bars represent mean ± SD of three biological replicates samples; *P < 0.05 (one-way ANOVA, Bonferroni corrected).

Our combined data support the idea that glucose acutely serves as a substrate to maintain oxygen consumption through increased PDH activity. As increased PDH activity can correlate with increased *de novo* lipogenesis^30,31^, we aimed to establish whether glucose indeed also enters lipogenesis. To do this, we examined the labelling pattern of the three most abundant triglycerides (TG)—TG(48:3), TG(50:3) and TG(54:3)—after incubation with [U-^13^C]-glucose using our lipidomics platform^34,35^. Glucose can contribute to TG synthesis by (1) producing glycerol-3-phosphate via glycolysis and thereby providing the TG backbone^36^; and/or (2) acetyl-CoA generation required for *de novo* fatty acid synthesis (Fig. 6A)^19,37^. We analysed the TG species of vehicle or CL316,243-stimulated cells for after 1 and 6 h (Fig. 6B**–**D). The most abundant TGs followed a similar labelling pattern, which was particularly enriched with ^13^C_3_ indicating incorporation of three ^13^C atoms likely through glycerol. This effect was even more pronounced in activated brown adipocytes (Fig. 6B**–**D). The incorporation of ^13^C_2_-labeled acetyl-CoA—after malonyl-CoA conversion and incorporation leading to ^13^C_[n**×**2]_-labelled TG —was relatively minor (Fig. 6B**–**D). Altogether, our results suggest that short-term β3-AR stimulation specifically induces glycolysis and glucose oxidation which is mediated by increased PDH activity. While the overall increased TCA cycle activity sustains increased respiration rates, the increased flux through PDH does not seem to induce *de novo* lipogenesis as glucose-derived acetyl-CoA is sparsely incorporated in TGs. Rather the increased glycolytic flux favours glycerol production required for esterification of fatty acids.

## Discussion

Brown adipose tissue activation is classically accompanied by increased uptake of glucose and lipids from the circulation. The induced uptake of glucose and lipids is such a key event that it is often used as a proxy for BAT activation^38,39^. The mechanism underlying simultaneous glucose and lipid uptake and utilization is not fully understood, despite being at the core of BAT function and its therapeutic potential. In the present study, we investigated substrate utilization by brown adipocytes during short-term β3-AR activation. We show that brown adipocytes indeed oxidize fatty acids and glucose simultaneously. β3-AR activation induced partitioned glucose utilization, i.e. allowing coinciding glucose oxidization and glucose utilization for alternative metabolic pathways, such as glycerol synthesis.

It has been suggested that the purpose of the increased influx of glucose is not to meet the direct increase in energy demand. Rather, it may be utilized as a building block for lipid synthesis to replenish the lipid pool for later usage^20,39^. Our data indicate that glucose is the second preferred substrate, but glucose oxidation is clearly induced upon β3-AR activation. In mice, glucose uptake and glycolysis are upregulated during BAT activation presumably to compensate for the loss of ATP due to UCP1-mediated uncoupling^40^. During short-term activation, the increased glycolytic rate did not only give rise to increased lactate levels (Fig. 3C), but also a considerable amount of glucose was oxidized into CO_2_ and thereby contributed to uncoupled respiration (Fig. 5D). It has been recently shown that UCP1 KO mice have diminished uncoupled respiration after β3-AR activation but glucose uptake and glycolysis were stimulated to similar extent as in the WT^41,42^. It is therefore likely that next to glucose oxidation, β3-AR stimulation also induces glucose metabolism to partly channel glucose to alternative pathways. Proposed alternative pathways are the pentose phosphate pathway, glycerol synthesis and *de novo* fatty acid synthesis^20–22^. We observed only a transient increase in PPP activity and therefore focused on TGs and fatty acid synthesis.

β-adrenergic signalling induces lipolysis via hormone sensitive lipase and adipose triglyceride lipase^43^. Genetic and pharmacological inhibition of lipolysis hampers UCP1-mediated uncoupling^44^, and thereby promotes cold intolerance in mice^17,45,46^ and humans^13^. With this in mind, it was postulated that fatty acids derived from intracellular lipid droplets are the main fuel source for brown adipocytes that must be continuously replenished. Our lipidomics analysis shows that incubation with [U-^13^C]-glucose predominantly gives rise to ^13^C_3_-labeled TGs. This is supportive of glucose conversion into glycerol which is subsequently used for TG synthesis^36^. This is in contrast to previous suggestions that glucose is primarily ending up in complex lipids through *de novo* fatty acid synthesis^19^. Such *de novo* lipogenesis would require glucose conversion to acetyl-CoA, after which these two-carbon units are utilized as the building blocks to elongate fatty acid chains. If such a scenario were true, we would have found TGs with two, four, six or more even ^13^C-labeled TGs (Fig. 6A). This is not the case suggesting that glucose is primarily entering the TG pool via glycerol, even though it is still possible that differences in growth phase, glucose concentration, intracellular lipid content, or specific cellular context affects the metabolic phenotype in BAT^47,48^.

Very recently, the principle that lipolysis is required for BAT activation has been challenged^49,50^. The loss of thermoregulation upon whole body or adipose-specific loss of lipolysis underlines the importance of fatty acids as a substrate for thermogenesis^15,17,45,46^. However, by selectively inhibiting BAT lipolysis only, it was shown that BAT can be very flexible in its substrate utilization leading to increased uptake of circulating substrates. The increased uptake of exogenous substrates was especially induced during cold exposure or specific β3-AR stimulation^49,50^. The fact that T37i brown adipocytes also predominantly exhibited fatty acid oxidation in the absence of lipolysis induced by β3-AR activation suggests metabolic plasticity. We have seen other modes of plasticity of brown adipocyte metabolism as well. When using a combination of inhibitors that prevent glucose and fatty acid oxidation, we found an increase in glutamine oxidation. Similarly, when glutamine and fatty acid oxidation were inhibited, glucose oxidation was elevated. This flexibility in substrate utilization was induced after β3-AR activation. The loss of fatty acid oxidation and the rise of intracellular fatty acids can activate peroxisome proliferator activated receptor (PPAR) signaling^51^, and thereby contribute to metabolic plasticity to meet the increase energy demands during BAT activation.

Glucose metabolism is regulated at multiple levels. Pyruvate is a key metabolite in glucose metabolism as it lies at the crossroads of glycolysis and TCA cycle activity. Pyruvate is an important substrate to maintain the high respiration rates during uncoupled respiration. Inhibition of pyruvate entrance into mitochondria has previously shown to attenuate noradrenaline-induced uncoupled respiration^52^. Likewise, inhibition of glycolysis and thus the reduction of pyruvate levels attenuated uncoupled respiration after β3-AR stimulation (Fig. 3A). Pyruvate supports TCA cycle activity through decarboxylation by PDH or carboxylation by PC^53^. Our metabolic flux analysis estimated an increase in flux through both enzymes. PDH is considered as a gatekeeper for glucose utilization and energy metabolism^53,54^. PDH senses intermediate metabolites —such as pyruvate and acetyl-CoA— and the NAD^+^/NADH redox state to control glucose metabolism and maintain optimal energy homeostasis^32^. In proliferating cells, genetic silencing of PDH could be compensated by increased uptake of lipids and increased glutamine utilization that is alternatively metabolized to contribute to lipid synthesis^54^. This underlines the plasticity of cellular metabolism, and it will be interesting to elucidate to what extent PDH is the main player involved in the partitioning of glucose metabolism between (a) full oxidation, and/or (b) glycerol and lipid synthesis. In the acute phase, we observed an induction of PDH activity upon β3-AR stimulation resulting in increased pyruvate oxidation which was similar to the maximal level reached by stimulation with PDH agonist DCA. Activating PDH may therefore favour BAT metabolism to allow optimal thermogenesis.

Collectively, our data demonstrate that glycolysis and glucose oxidation contribute to uncoupled respiration, despite that fatty acids are the main fuel for brown adipocytes. Glucose metabolism appeared to be heavily regulated upon β3-AR stimulation. We identified PDH as a gatekeeper for glucose utilization that directs most glucose towards oxidation to maintain uncoupled respiration. The accompanying increase of PC activity to convert pyruvate to oxaloacetate replenishes the TCA cycle during this high PDH activity. Oxaloacetate could also act as a precursor for glyceroneogenesis, which is the formation of glycerol-3-phosphate via phosphoenolpyruvate carboxykinase activity^36,55^, and thereby allow part of the glucose to be directed to glycerol synthesis and storage of free fatty acids as TGs in lipid droplets as substrates for future use. Pharmacological targeting of the PDH holds potential as a novel strategy to potentiate BAT function.

## Material and Methods

### Cell culture of T37i brown adipocytes and Oil-Red-O staining

T37i cells were cultured and differentiated as described previously^24,25^. In brief, cells were kept in maintenance culture in DMEM/F12 Glutamax supplement (Life technologies) containing 10% FBS (BioWhittaker), 100 IU/mL penicillin and 10 mg/mL streptomycin (Life technologies) until passage 37. For differentiation, cells were kept at complete confluency and after two days 2 nM triiodothyronine (Sigma-Aldrich) and 2 µM insulin (Sigma-Aldrich) was added to the medium for 9 days. During differentiation, medium was replaced every two days and cells were used for experiments between differentiation day 10–12. Oil-Red-O (Sigma-Aldrich) staining was performed to evaluate lipid droplet accumulation. In short, cells were washed with PBS and fixed in 10% (v/v) formalin for 1 h, rinsed in 60% (v/v) isopropanol for 5 min, and stained with filtered 60% Oil-Red-O solution for 15 min. Excess of Oil Red O was removed and cells were maintained in demineralized water during imaging.

### Gene expression and protein content in T37i cells

Total RNA was isolated with TRIreagent (Sigma-Aldrich) according to the manufacturer’s instructions including addition of DNase treatment (Promega). cDNA synthesis was performed with 1 µg RNA using the QuantiTect Reverse Transcription Kit (QIAGEN). LightCycler 480 SYBR Green I Master (Roche) was used for qPCR analysis, primers are listed in Table 1. Data were analysed with Light Cycler 480 software release 1.5 and LinRegPCR version 2015.3, as previously described^56^.

**Table 1:** Primers.

Gene	Accession	Forward primer	Reverse primer
*ACTB*	NM_007393	AACCGTGAAAAGATGACCCAGAT	CACAGCCTGGATGGCTACGTA
*CIDEA*	NM_007702	ATCACAACTGGCCTGGTTACG	TACTACCCGGTGTCCATTTCT
*DIO2*	NM_010050	GGCCGTCGGTCCTTCCTT	TCCCAGCTGTGTACATGCCTCAAT
*GAPDH*	NM_008084	GGGGCTGGCATTGCTCTCAA	TTGCTCAGTGTCCTTGCTGGGG
*PPIA*	NM_008907	CAAATGCTGGACCAAACACAA	GCCATCCAGCCATTCAGTCT
*PRDM16*	NM_027504	CAGCACGGTGAAGCCATTC	GCGTGCATCCGCTTGTG
*UCP1*	NM_009463	ACGTCCCCTGCCATTTACTGTCA	GGCCGTCGGTCCTTCCTT

For protein extraction, T37i cells were lysed in RIPA buffer (50 mM Tris-HCl pH 7.4, 150 mM NaCl, 0.1% (w/v) sodium dodecyl sulfate, 0.5% (w/v) sodium deoxycholate, 1% (v/v) Triton X-100) with addition of Complete mini protease inhibitor cocktail (Roche) and Phosphatase Inhibitor Cocktail 2 and 3 (Sigma-Aldrich). Samples were lysed by tip sonication and protein concentration was measured using the BCA protein assay kit (Pierce). For immunoblot analysis, lysates were diluted in NuPAGE LDS Sample Buffer and Sample Reducing Agent (Life Technologies) and heated to 70 °C. Protein extracts were separated on pre-cast NuPAGE 4–12% gradient Bis-Tris gels (Life Technologies), and transferred to a nitrocellulose membrane. Membranes were blocked with 3% BSA (in PBS containing 0.1% (v/v) Tween-20), and incubated overnight at 4 °C with the following primary antibodies: total PDHE1α (#ab67592, Abcam), phospho-Ser232 PDHE1α (#AP1063, Calbiochem) and HSP60 (#4870, Cell Signaling). The immunoreactive bands were detected with HRP-linked secondary antibodies (Goat anti-rabbit, Goat anti-mouse, DAKO) and ECL prime western blotting detection reagent (Amersham) and imaged with the ImageQuant LAS4000 (GE Healthcare). Quantification of bands was performed using Bio-Rad Quantity one 4.6.6 software.

### Oxygen consumption

Oxygen consumption rate (OCR) and extracellular acidification rate (ECAR) was measured using the Seahorse XF96 analyzer (Seahorse Bioscience). T37i cells were plated at differentiation day 9 in 96-well Seahorse plates at a density of 60,000 cells per well and incubated overnight under normal cell culture conditions. The following day, medium was replaced by DMEM (Sigma, D5030) containing 17.5 mM glucose (Sigma-Aldrich), 1 mM sodium pyruvate (Lonza), and 2 mM L-Glutamine (Life technologies). Basal respiration was measured three times followed by six measurements after addition of 10 µM β3-AR agonist CL316,243 (Tocris) to induce brown adipocyte activation. ATP-coupled respiration and the maximal respiration were determined by the addition of 1.5 µM oligomycin and 1.5 µM FCCP (Sigma-Aldrich), respectively. OCR was corrected for non-mitochondrial respiration determined by simultaneous addition of 2.5 µM antimycin A and 1.25 µM rotenone (Sigma-Aldrich).

Glycolytic function was determined in DMEM medium (Sigma, D5030) containing 2 mM L-Glutamine (Life technologies) according to Seahorse XF Glycolysis stress test manufacturer instructions. In brief, basal ECAR was measured three times followed by six measurements after sequential addition of (a) 10 µM CL316,243 or vehicle (medium), (b) 10 mM glucose, (c) 1.5 µM oligomycin and (d) 100 mM 2-deoxy-glucose (Sigma-Aldrich).

Substrate dependency and reserve capacity were determined according the Seahorse XF Mito Fuel Flex Test user guide protocol. Briefly, after measuring basal OCR and after addition of 10 µM CL316,243 or vehicle; 100 µM POCA (sodium 2-[5-(4-chlorophenyl)-pentyl]oxirane-2-carboxylate), a CPT1 inhibitor (kind gift from BYK Gulden Pharmazeutica); 3 µM BPTES (Bis-2-(5-phenylacetamido-1,3,4-thiadiazol-2-yl)ethyl sulfide), a glutaminase inhibitor (Sigma-Aldrich) and 2 µM UK5099, an inhibitor of the mitochondrial pyruvate transporter (Sigma-Aldrich) were subsequently injected and OCR was determined six times. Data were analysed using Seahorse XF Mito Fuel Flex test report data analysis.

All ECAR and OCR values were adjusted for cell input using the CyQUANT Cell Proliferation Assay Kit (Thermo Fischer Scientific) according to the manufacturer’s instruction. The final measurement point after each compound addition was always used for quantification.

### Pyruvate oxidation

Pyruvate oxidation was determined by measuring the release of ^14^CO_2_ from [1-^14^C]-pyruvate^57^. At differentiation day 9, cells were seeded at a density of 500,000 cells in a glass liquid scintillation vial under normal cell culture conditions. The following day, cells were washed twice with DPBS prior to a 1 h incubation in Dulbecco’s PBS (Life Technologies) supplemented with 500 µM [1-^14^C]-pyruvate (specific activity: 0.55 mCi/mmol) (Perkin Elmer) combined with 10 µM CL316,243 or vehicle (PBS). A centre well containing 2 M NaOH was placed to trap CO_2_. After 1 h of shaking at 37 °C, medium was acidified with 2.6 M perchloric acid to stop the reaction. After 3 h of trapping, the ^14^CO_2_ collected in the centre well was counted by liquid scintillation. Pyruvate oxidation flux was determined by the amount of pyruvate oxidized to CO_2_ normalized to protein content.

### Isotopic labelling of polar metabolites

Cells were differentiated in 6-well plates. At differentiation day 10, a time course incubation was started in DMEM without glucose, pyruvate, glutamine and phenol red (Life technologies) with addition of 17.5 mM [U-^13^C]-glucose (Cambridge Isotope Laboratories)in combination with 10 µM CL316,243 or vehicle (medium). Samples were harvested by two-phase methanol-water/chloroform extraction as described^58^. Briefly, medium was removed, cells were washed twice with ice-cold 0.9% NaCl, and metabolism was quenched by the addition of 1 mL ice-cold methanol-water (1:1, v/v). Cells were removed from the well by scraping and collected in a centrifuge tube. One mL of chloroform was added to the mixture, followed by tip sonication and centrifugation at 10,000 × *g* for 10 min. The organic phase of the extraction was collected for lipidomic analysis (see below). After collection of the aqueous phase, the organic phase and insoluble pellet were re-extracted with 1 mL methanol-water (1:1, v/v). The aqueous phases of both extractions were collected and evaporated. The metabolite residue was dissolved in 100 µL 60% (v/v) methanol and analyzed by ultra-high-pressure liquid chromatography system (Thermo Scientific) with a SeQuant ZIC-cHILIC column (100 × 2.1 mm, 3 µm particle size, Merck, Darmstadt, Germany) coupled to a Thermo Q Exactive Plus Orbitrap mass spectrometer (Thermo Scientific). The column was kept at 15 °C and the flow rate was 0.250 mL/min. The mobile phase was composed of (A) 9/1 acetonitrile/water with 5 mM ammonium acetate; pH 6.8 and (B) 1/9 acetonitrile/water with 5 mM ammonium acetate; pH 6.8, respectively. The LC gradient program was: beginning with 100% (A) hold 0–3 min; ramping 3–20 min to 36% (A); ramping from 20–24 min to 20% (A); hold from 24–27 min at 20% (A); ramping from 27–28 min to 100% (A); and re-equilibrate from 28–35 min with 100% (A). Data were acquired in full-scan negative ionization mode. Data interpretation was performed using the Xcalibur software (Thermo Scientific). ^13^C enrichment was calculated based on mass distribution isotopomer analysis (MIDA), all results were corrected for their natural ^13^C abundance by solving the associated set of linear equations for each metabolite using non-negative least squares^59^.

### Non-stationary metabolic flux analysis

Non stationary metabolic flux analysis was based on the algorithm described in^60^. In short, the carbon transition model of the TCA cycle (Table 2) was converted into a set of linear ordinary differential equations describing the time dynamics of the corresponding Elementary Metabolite Units (EMUs) using an in-house pipeline developed in MATLAB (The MathWorks, Natick, USA). Subsequently, the flux and metabolite concentrations were estimated by minimizing the sum of the squared residuals of the measured MID time data with respect to the model prediction using the Levenberg-Marquardt algorithm.

**Table 2:** Carbon transition model used for non-stationary metabolic flux analysis.

Substrate	Reaction	Product
citrate(abcdef)	→	alpha-ketoglutarate(abdef) + CO_2_(c)
pyruvate(abc)	→	acetyl-CoA(ab) + CO_2_(c)
acetyl-CoA(ab) + fumarate_malate(cdef)	→	citrate(bacdef)
alpha-ketoglutarate(abcde)	→	succinate(abcd) + CO_2_(e)
alpha-ketoglutarate(abcde)	→	succinate(dcba) + CO_2_(e)
succinate(abcd)	→	fumarate_malate(abcd)
fumarate_malate(abcd)	→	succinate(abcd)
pyruvate(abc) + CO_2_(d)	→	fumarate_malate(dabc)
pyruvate(abc) + CO_2_(d)	→	fumarate_malate(cbad)

### Isotopic labelling of lipids

The aforementioned organic phase was processed according to the lipidomic sample preparation work flow described previously^35^, with minor modifications. A mixture of internal standards for multiple lipid classes was added to 250 μL of the organic phase. The internal standard mixture (all lipids from Avanti Polar Lipids) contained the following lipid concentrations: 7.5 nmol of phosphatidylserine PS(14:0)_2_; 3.0 nmol of phosphatidylcholine PC(14:0)_2_ and sphingomyelin SM(d18:1/12:0); 0.75 nmol of diacylglycerol DAG(14:0)_2_, triacylglycerol TAG(14:0)_3_, cholesteryl ester CE(14:0), phosphatidylinositol PI(14:0)_2_, phosphatidylethanolamine PE(14:0)_2_, phosphatidic acid PA(14:0)_2_, lysophosphatidylcholine LPC(14:0); 0.3 nmol of bis(monoacylglycero)phosphate BMP(14:0)_2_; 0.15 nmol of cardiolipin CL(14:0)_4_, phosphatidylglycerol PG(14:0)_2_, lysophosphatidylglycerol LPG(14:0), lyso-phosphatidylethanolamine LPE(14:0) and lysophosphatidic acid LPA(14:0). After addition of internal standard mixture, the organic phase was evaporated under nitrogen flow. The lipid extract was dissolved in 100 μL of methanol/chloroform (1:1, v/v) and analyzed as described^35^. In brief, samples were injected onto a LiChrospher silica-60 (5 μm) HPLC column (Merck) coupled to a Thermo Q Exactive Plus Orbitrap mass spectrometer (Thermo Scientific). Data were acquired in negative and positive scan mode and processed using in-house developed metabolomics pipeline written in the R programming language. The identified peaks were normalized to the intensity of the internal standard for each lipid class. ^13^C enrichment in selected lipids was calculated based on MIDA, all results were corrected for their natural ^13^C abundance by solving the associated set of linear equations for each metabolite using non-negative least squares^59,60^.

### Statistical analysis

Groups were compared using a two-tailed Student’s *t*-test or one-way ANOVA with post hoc Bonferroni correction using GraphPad Prism (GraphPad Software v5.0). *P* < 0.05 was considered statistically significant. Data are displayed as mean ± SD or SEM, as indicated in the figure legends.

## Electronic supplementary material

Supplementary Information
