# Supplementary material for: Pyruvate dehydrogenase complex plays a central role in brown adipocyte energy expenditure and fuel utilization during short-term beta-adrenergic activation
Source: Sci Rep. 2018 Jun 22;8:9562. doi: 10.1038/s41598-018-27875-3 (PMC6015083; doi:10.1038/s41598-018-27875-3)
Supplement: Supplementary file 1 — Supplementary Information [file 41598_2018_27875_MOESM1_ESM.docx]

**SUPPLEMENTARY INFORMATION**

**Pyruvate dehydrogenase complex plays a central role in brown adipocyte energy expenditure and fuel utilization during short-term beta-adrenergic activation**

Ntsiki M. Held^1,#^, Eline N. Kuipers^2,3,#^, Michel van Weeghel^1^, Jan Bert van Klinken^3,4^, Simone W. Denis^1^, Marc Lombès^5^, Ronald J. Wanders^1^, Frédéric M. Vaz^1^, Patrick C.N. Rensen^2,3^, Arthur J. Verhoeven^1^, Mariëtte R. Boon^2,3^ and Riekelt H. Houtkooper^1^*

*^1^Laboratory Genetic Metabolic Diseases, Academic Medical Center, 1105 AZ Amsterdam, The Netherlands*

*^2^Department of Medicine, Division of Endocrinology, Leiden University Medical Center, 2333 ZA Leiden, the Netherlands*

*^3^Einthoven Laboratory of Experimental Vascular Medicine, Leiden University Medical Center,* ***2333 ZA*** *Leiden, The Netherlands*

*^4^Department of Human Genetics, Leiden University Medical Center,* ***2333 ZA*** *Leiden, The Netherlands*

*^5^Institut National de la Santé et de la Recherche Médicale, Unité 1185, Le Kremlin-Bicêtre, UMR_S 1185, Fac Med Paris Sud, Université Paris-Saclay, France*

***Corresponding author:** Riekelt Houtkooper; [r.h.houtkooper@amc.nl](mailto:r.h.houtkooper@amc.nl); Laboratory Genetic Metabolic Diseases, Academic Medical Center, 1105 AZ Amsterdam, The Netherlands; +31 20563927.

**^#^** Contributed equally

**Table S1** Fractional contribution of ^13^C- glucose corrected for natural abundance in pentose phosphate pathway (PPP) and glycolytic intermediates upon vehicle or CL316,243 time course stimulation

|  | **Metabolite** | **0.5 h** | | **1 h** | | **2 h** | | **4 h** | | **7 h** | |
| --- | --- | --- | --- | --- | --- | --- | --- | --- | --- | --- | --- |
|  |  | **Veh** | **CL** | **Veh** | **CL** | **Veh** | **CL** | **Veh** | **CL** | **Veh** | **CL** |
| **PPP** | R5P | 0.118 | 0.049 | 0.135 | 0.057 | 0.173 | 0.140 | 0.273 | 0.277 | 0.481 | 0.430 |
|  | ER-4P | 0.800 | 0.765 | 0.756 | 0.930 | 0.917 | 0.956 | 0.947 | 1.000 | 0.996 | 0.977 |
|  | SED-7P | 0.815 | 0.688 | 0.803 | 0.756 | 0.922 | 0.843 | 0.939 | 0.928 | 0.954 | 0.956 |
| **Glycolysis** | G6P | 0.825 | 0.682 | 0.775 | 0.767 | 0.881 | 0.801 | 0.881 | 0.903 | 0.915 | 0.894 |
|  | G3P/DHAP | 0.746 | 0.624 | 0.716 | 0.643 | 0.818 | 0.841 | 0.884 | 0.815 | 0.976 | 0.916 |
|  | BPG | 0.755 | 0.532 | 0.681 | 0.671 | 0.868 | 0.789 | 0.911 | 0.940 | 0.986 | 0.949 |
|  | PG | 0.751 | 0.507 | 0.686 | 0.635 | 0.872 | 0.777 | 0.872 | 0.940 | 0.966 | 0.945 |
|  | PEP | 0.721 | 0.528 | 0.669 | 0.638 | 0.847 | 0.768 | 0.893 | 0.930 | 0.942 | 0.947 |
|  | Pyruvate | 0.771 | 0.694 | 0.742 | 0.762 | 0.859 | 0.830 | 0.891 | 0.900 | 0.916 | 0.916 |
|  | Lactate | 0.776 | 0.740 | 0.775 | 0.795 | 0.856 | 0.840 | 0.868 | 0.886 | 0.911 | 0.902 |

**Table S2 related to Figure 4** Fractional distribution of isotopologues corrected for natural abundance used to determine metabolite labeling as shown in Figure 4A.

|  | **Fractional distribution** | | | | | | | | | |
| --- | --- | --- | --- | --- | --- | --- | --- | --- | --- | --- |
|  | **0.5 h** | | **1 h** | | **2 h** | | **4 h** | | **7 h** | |
| **Metabolite** | **Veh** | **CL** | **Veh** | **CL** | **Veh** | **CL** | **Veh** | **CL** | **Veh** | **CL** |
| Citrate ^13^C_0_ | 0.579 | 0.451 | 0.573 | 0.382 | 0.454 | 0.305 | 0.378 | 0.256 | 0.303 | 0.199 |
| Citrate ^13^C_1_ | 0.003 | 0.015 | 0.011 | 0.033 | 0.019 | 0.032 | 0.020 | 0.033 | 0.020 | 0.032 |
| Citrate ^13^C_2_ | 0.126 | 0.191 | 0.098 | 0.184 | 0.159 | 0.186 | 0.151 | 0.176 | 0.131 | 0.178 |
| Citrate ^13^C_3_ | 0.096 | 0.138 | 0.094 | 0.157 | 0.113 | 0.158 | 0.141 | 0.167 | 0.123 | 0.167 |
| Citrate ^13^C_4_ | 0.060 | 0.083 | 0.067 | 0.093 | 0.083 | 0.119 | 0.101 | 0.125 | 0.125 | 0.150 |
| Citrate ^13^C_5_ | 0.116 | 0.102 | 0.120 | 0.122 | 0.146 | 0.155 | 0.173 | 0.189 | 0.230 | 0.209 |
| Citrate ^13^C_6_ | 0.019 | 0.021 | 0.037 | 0.030 | 0.026 | 0.044 | 0.034 | 0.054 | 0.068 | 0.065 |
| α-Ketoglutarate ^13^C_0_ | 0.780 | 0.659 | 0.718 | 0.571 | 0.550 | 0.439 | 0.411 | 0.338 | 0.278 | 0.264 |
| α-Ketoglutarate ^13^C_1_ | 0.013 | 0.032 | 0.023 | 0.050 | 0.046 | 0.063 | 0.061 | 0.069 | 0.056 | 0.061 |
| α-Ketoglutarate ^13^C_2_ | 0.132 | 0.175 | 0.166 | 0.202 | 0.235 | 0.236 | 0.273 | 0.253 | 0.278 | 0.254 |
| α-Ketoglutarate ^13^C_3_ | 0.044 | 0.070 | 0.055 | 0.092 | 0.093 | 0.114 | 0.122 | 0.138 | 0.146 | 0.147 |
| α-Ketoglutarate ^13^C_4_ | 0.020 | 0.041 | 0.026 | 0.055 | 0.050 | 0.093 | 0.086 | 0.127 | 0.153 | 0.171 |
| α-Ketoglutarate ^13^C_5_ | 0.011 | 0.023 | 0.013 | 0.031 | 0.026 | 0.054 | 0.047 | 0.076 | 0.088 | 0.104 |
| Succinate ^13^C_0_ | 0.755 | 0.632 | 0.697 | 0.547 | 0.554 | 0.448 | 0.587 | 0.398 | 0.367 | 0.361 |
| Succinate ^13^C_1_ | 0.015 | 0.048 | 0.026 | 0.068 | 0.051 | 0.077 | 0.047 | 0.076 | 0.060 | 0.067 |
| Succinate ^13^C_2_ | 0.145 | 0.203 | 0.184 | 0.240 | 0.247 | 0.265 | 0.210 | 0.275 | 0.286 | 0.266 |
| Succinate ^13^C_3_ | 0.050 | 0.056 | 0.048 | 0.062 | 0.076 | 0.079 | 0.076 | 0.091 | 0.116 | 0.117 |
| Succinate ^13^C_4_ | 0.035 | 0.061 | 0.044 | 0.083 | 0.073 | 0.130 | 0.080 | 0.160 | 0.171 | 0.190 |
| Fumarate ^13^C_0_ | 0.590 | 0.500 | 0.557 | 0.410 | 0.440 | 0.333 | 0.360 | 0.279 | 0.256 | 0.268 |
| Fumarate ^13^C_1_ | 0.022 | 0.054 | 0.030 | 0.067 | 0.043 | 0.069 | 0.052 | 0.069 | 0.056 | 0.065 |
| Fumarate ^13^C_2_ | 0.110 | 0.160 | 0.113 | 0.184 | 0.149 | 0.197 | 0.172 | 0.198 | 0.205 | 0.200 |
| Fumarate ^13^C_3_ | 0.242 | 0.226 | 0.260 | 0.258 | 0.313 | 0.286 | 0.357 | 0.323 | 0.365 | 0.340 |
| Fumarate ^13^C_4_ | 0.035 | 0.060 | 0.040 | 0.081 | 0.055 | 0.115 | 0.059 | 0.132 | 0.118 | 0.127 |
| Malate ^13^C_0_ | 0.625 | 0.510 | 0.573 | 0.419 | 0.480 | 0.365 | 0.401 | 0.316 | 0.267 | 0.284 |
| Malate ^13^C_1_ | 0.047 | 0.110 | 0.066 | 0.119 | 0.077 | 0.111 | 0.090 | 0.098 | 0.092 | 0.084 |
| Malate ^13^C_2_ | 0.087 | 0.128 | 0.092 | 0.153 | 0.125 | 0.165 | 0.147 | 0.172 | 0.203 | 0.176 |
| Malate ^13^C_3_ | 0.211 | 0.200 | 0.235 | 0.237 | 0.272 | 0.261 | 0.311 | 0.299 | 0.337 | 0.348 |
| Malate ^13^C_4_ | 0.030 | 0.053 | 0.034 | 0.072 | 0.046 | 0.098 | 0.051 | 0.115 | 0.101 | 0.108 |
| Glutamate ^13^C_0_ | 0.784 | 0.666 | 0.722 | 0.579 | 0.556 | 0.451 | 0.419 | 0.347 | 0.287 | 0.272 |
| Glutamate ^13^C_1_ | 0.013 | 0.031 | 0.024 | 0.049 | 0.047 | 0.063 | 0.062 | 0.070 | 0.057 | 0.062 |
| Glutamate ^13^C_2_ | 0.129 | 0.171 | 0.163 | 0.201 | 0.232 | 0.233 | 0.269 | 0.251 | 0.276 | 0.254 |
| Glutamate ^13^C_3_ | 0.044 | 0.069 | 0.053 | 0.089 | 0.091 | 0.111 | 0.120 | 0.135 | 0.145 | 0.145 |
| Glutamate ^13^C_4_ | 0.020 | 0.040 | 0.025 | 0.052 | 0.049 | 0.090 | 0.085 | 0.123 | 0.149 | 0.166 |
| Glutamate ^13^C_5_ | 0.011 | 0.023 | 0.013 | 0.030 | 0.026 | 0.052 | 0.045 | 0.073 | 0.086 | 0.101 |

**Figure S1. Original Western blot images, related to Figure 6B.**

**
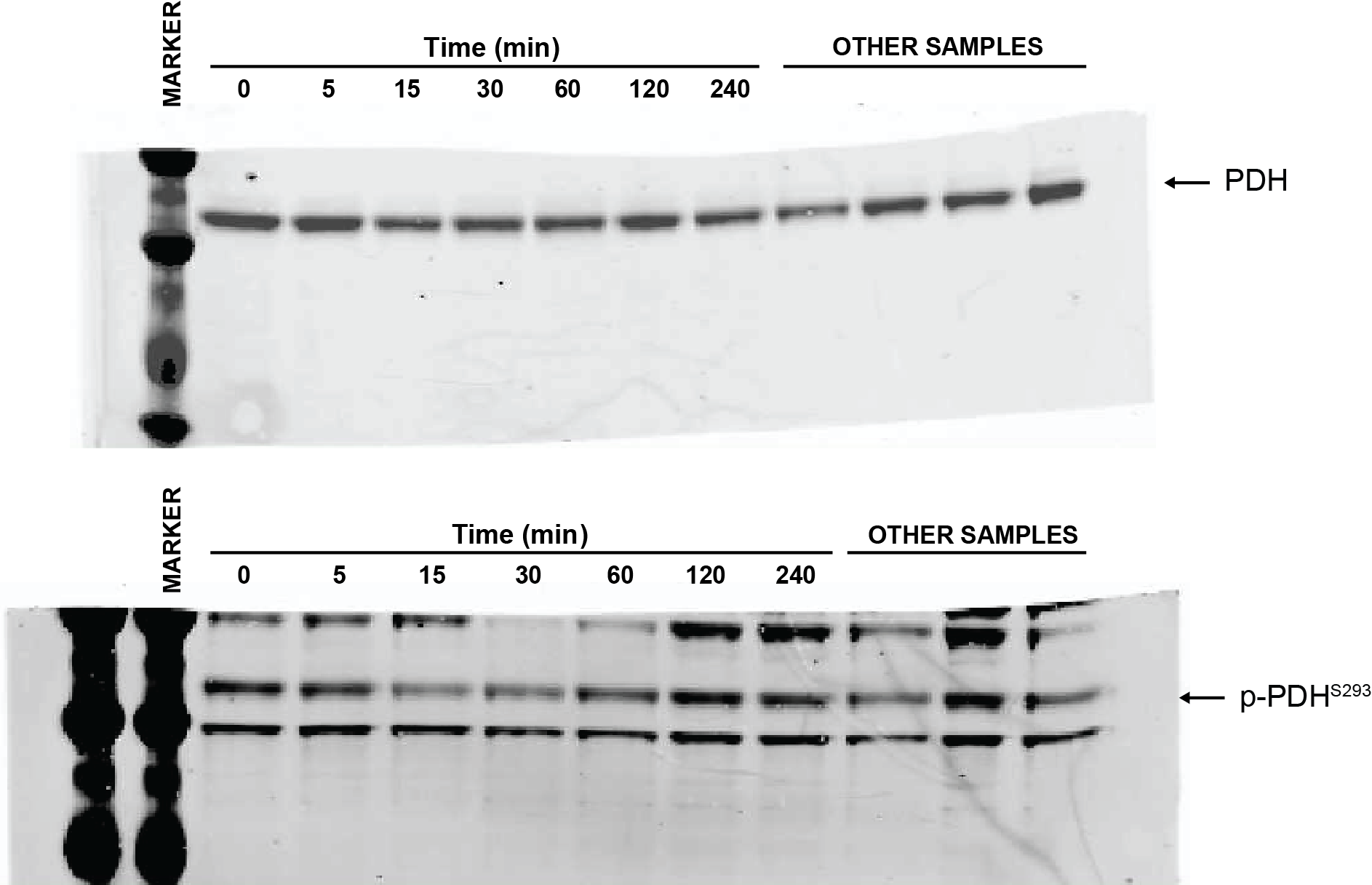
**
